# Tolerance and metabolic response of *Pseudomonas taiwanensis* VLB120 towards biomass hydrolysate-derived inhibitors

**DOI:** 10.1186/s13068-018-1192-y

**Published:** 2018-07-19

**Authors:** Gossa G. Wordofa, Mette Kristensen

**Affiliations:** Novo Nordisk Foundation Center for Biosustainability, Technical University of Denmark, 2800 Lyngby, Denmark

**Keywords:** Biomass hydrolysate inhibitors, Inhibitor tolerance, Metabolomics, *Pseudomonas taiwanensis*

## Abstract

**Background:**

Bio-conversion of lignocellulosic biomass to high-value products offers numerous benefits; however, its development is hampered by chemical inhibitors generated during the pretreatment process. A better understanding of how microbes naturally respond to those inhibitors is valuable in the process of designing microorganisms with improved tolerance. *Pseudomonas taiwanensis* VLB120 is a natively tolerant strain that utilizes a wide range of carbon sources including pentose and hexose sugars. To this end, we investigated the tolerance and metabolic response of *P. taiwanensis* VLB120 towards biomass hydrolysate-derived inhibitors including organic acids (acetic acid, formic acid, and levulinic acid), furans (furfural, 5-hydroxymethylfurfural), and phenols (vanillin).

**Results:**

The inhibitory effect of the tested compounds varied with respect to lag phase, specific growth rate, and biomass yield compared to the control cultures grown under the same conditions without addition of inhibitors. However, *P. taiwanensis* was able to oxidize vanillin and furfural to vanillic acid and 2-furoic acid, respectively. Vanillic acid was further metabolized, whereas 2-furoic acid was secreted outside the cells and remained in the fermentation broth without further conversion. Acetic acid and formic acid were completely consumed from the fermentation broth, while concentration of levulinic acid remained constant throughout the fermentation process. Analysis of free intracellular metabolites revealed varying levels when *P. taiwanensis* VLB120 was exposed to inhibitory compounds. This resulted in increased levels of ATP to export inhibitors from the cell and NADPH/NADP ratio that provides reducing power to deal with the oxidative stress caused by the inhibitors. Thus, adequate supply of these metabolites is essential for the survival and reproduction of *P. taiwanensis* in the presence of biomass-derived inhibitors.

**Conclusions:**

In this study, the tolerance and metabolic response of *P. taiwanensis* VLB120 to biomass hydrolysate-derived inhibitors was investigated. *P. taiwanensis* VLB120 showed high tolerance towards biomass hydrolysate-derived inhibitors compared to most wild-type microbes reported in the literature. It adopts different resistance mechanisms, including detoxification, efflux, and repair, which require additional energy and resources. Thus, targeting redox and energy metabolism in strain engineering may be a successful strategy to overcome inhibition during biomass hydrolysate conversion and lead to development of more robust strains.

**Electronic supplementary material:**

The online version of this article (10.1186/s13068-018-1192-y) contains supplementary material, which is available to authorized users.

## Background

*Pseudomonas taiwanensis* is an obligate aerobe, biofilm-forming organism that was isolated from soil at the Institute of Microbiology, University of Stuttgart, Germany [1–4]. It can thrive in diverse habitats, and is known for its ability to colonize soil and participate in soil biochemical processes [5, 6]. The potential of *P. taiwanensis* for the degradation and bioremediation of a wide variety of chemicals, including natural and synthetic compounds, such as caprolactam [7], naphthalene [8], and toluene, has attracted a great research interest [4]. Furthermore, the strain utilizes a wide range of organic molecules as carbon sources including pentose/hexose sugars and aromatic hydrocarbons [2].

Unlike other industrially relevant *Pseudomonas putida* strains, such as *P. putida* KT2440, *P. putida* DOT-T1E, and *P. putida* S12, *P. taiwanensis* VLB120 is the only known *Pseudomonas* strain that is able to utilize xylose as the sole carbon and energy source without any genetic modifications [2]. These remarkable features of *P. taiwanensis* emphasize its potential for the production of high-value products, such as n-butanol from low-cost renewable feedstocks through rational metabolic engineering as shown in a variety of heterologous microorganisms, including those cultivated aerobically such as *P. putida* [9].

While the physiology of *P. taiwanensis* VLB120 matches the basic requirements for growth on biomass hydrolysate, its exposure to biomass hydrolysate-derived inhibitors including acetic acid, formic acid, levulinic acid, furfural, 5-HMF, and vanillin has not yet been characterized. These compounds influence the growth of microorganisms in various ways, including DNA mutation, membrane disruption, intracellular pH drop, and other cellular targets [10, 11]. Therefore, understanding how *P. taiwanensis* metabolically respond to inhibitors and identifying which metabolic pathways and metabolites are involved can hasten the development of the strain to a production strain. These information can also be used to design other robust strains that are not able to grow on biomass hydrolysate naturally. Hence, the main aim of this work was to determine the tolerance and metabolic response of *P. taiwanensis* VLB120 toward the main inhibitory compounds present in lignocellulosic biomass hydrolysates.

## Methods

### Strain and culture mediums

*Pseudomonas taiwanensis* VLB120 was obtained from the Institute of Applied Microbiology, RWTH Aachen, Germany. The cell culture medium used on this study consisted of (L^−1^): 2.12-g NaH_2_PO_4_∙2H_2_O, 2-g (NH_4_)_2_SO_4_, 10-mg EDTA, 0.1-g MgCl_2_∙6H_2_O, 2-mg ZnSO_4_∙7H_2_O, 1-mg CaCl_2_∙2H_2_O, 5-mg FeSO_4_∙7H_2_O, 0.2 mg Na_2_MoO_4_∙2H_2_O, 0.2-mg CuSO_4_∙5H_2_O, 0.4-mg CoCl_2_∙6H_2_O, 1-mg MnCl_2_∙2H_2_O, and 4.5-g glucose as a carbon source [12]. Unless stated otherwise, all chemicals and reagents used in this study were purchased from Sigma-Aldrich (Chemical Co, USA).

### Inhibitors threshold concentration test

The inhibitor threshold concentration affecting growth was evaluated using the Growth Profiler 960 (EnzyScreen, Heemstede, The Netherlands). The inhibitory compounds were added into minimal medium supplemented with 4.5 g L^−1^ of glucose in different concentration levels. The media pH was adjusted to 7.0 ± 0.03 with 5 M of sodium hydroxide before inoculation. The same medium without inhibitory compounds was used as control.

Aerobic cultivations were carried out in 24-well clear bottom microplate (EnzyScreen, Heemstede, The Netherlands) working volume 750 µL at 30 °C, 225 rpm. The Growth Profiler was set to generate a scan of the plate every 20 min. Based on this scan, the Growth Profiler software was used to calculate the density of the cultures in each single well of a plate (green value; *G* value). A calibration curve was generated to convert the *G* values into optical density (OD) values. The following equation was obtained from the calibration curve and used throughout the study:

$$ {\text{OD}}_{{600\;{\text{nm}}}}\;        {\text{equivalent}} = 0.0158 \times G\;{\text{value}}^{1.304} . $$

### Bioreactor-batch growth experiment

Bioreactor-batch cultivations were performed to characterize the metabolic response of *P. taiwanensis* VLB120 under stress conditions. The experiments were performed in 1.3-L bioreactors (SARTORIOUS ^®^) with 0.5-L working volume. Cultures were inoculated at OD of approx. 0.05 and fermentation temperature, stirrer speed, and pH were set at 30 °C, 800 rpm, and 7.0, respectively. Cultures were supplied with air at a flow rate of 1 slpm, and minimum dissolved oxygen saturation level was 40%. The whole fermentation process was monitored by continuously measuring the CO_2_ percentage in the off-gas. All cultures were performed in triplicates and batch cultures were run for 24 h.

### Sample preparation for metabolome analysis

During bioreactor-batch growth experiments, supernatants were collected along the cultivation to quantify optical density at 600 nm (Spectrophotometer VWR UV-1600PC, USA) as well as extracellular metabolites. Samples for extracellular metabolite analysis were spun down at 10,000*g* for 5 min and stored at − 20 °C for further use. Samples for intracellular metabolite measurement were rapidly harvested (3 mL) with an electronic pipette at optical density of 0.4–0.6 (OD_600 nm_), and filtered with fast filtration system as described previously [13]. Immediately after the filtration process, quenching and extraction of metabolites were performed by adding 2 mL of 75% (v/v) boiling ethanol (70 °C) and 25 µL of fully labeled ^13^C cell extracts as an internal standard (IS) to the filtered cells and heated for 1 min. The cells were re-extracted by adding additional 1.5 mL of boiling ethanol at 70 °C. The samples were concentrated by evaporating the organic solvent for 5 h at 25 °C using a vacuum concentrator (SAVANT, SpeedVac, Thermo Fisher Scientific, San Diego, CA, USA) followed by lyophilization (LABCONCO, FreeZone, Kansas City, MO, USA) overnight at − 40 °C. All dried extracts were re-suspended in 250 μL of LC–MS grade water, which is compatible with the initial mobile phase of the LC–MS method and stored at − 80 °C until analysis.

### Measurement of inhibitors and extracellular metabolites

The concentration of inhibitors and extracellular metabolites was measured by high-performance liquid chromatography (HPLC). More specifically, quantification of furfural, 5-HMF, vanillin, and their corresponding acid in media was performed on a Dionex Ultimate 3000 HPLC equipped with a Supelco Discovery HS F5-3 HPLC column (150 × 2.1 mm × 3 µm) and a UV detector (260, 277, 304, and 210 nm). Samples (1 µL) were analyzed using a gradient method with mobile phase A: 10-mM ammonium formate, pH 3, and B: acetonitrile. A flow rate of 0.7 mL min^−1^ was used and the column was held at 30 °C. The program started with 5% of solvent B for 0.5 min and increased linearly to 60% over 5 min. The gradient was thereafter increased to 90% B over 0.5 min and kept at this condition for 2 min. Finally, returned to 5% B and equilibrated until 10 min.

Concentrations of glucose, gluconate, acetic acid, formic acid, and levulinic acid were determined using a Dionex Ultimate 3000 HPLC with an Aminex^®^ HPX-87X Ion Exclusion (300 × 7.8 mm) column (Bio-Rad, Hercules, CA) and RI-150 refractive index detector. Gluconate was measured by UV monitoring at 210 nm. The mobile phase consisted of 5-mM H_2_SO_4_, the flow rate was 0.6 mL min^−1^ and the column was kept at 60 °C. Samples were held at 5 °C during the analysis and 20-µL sample volume injected.

### Measurement of intracellular metabolites

Metabolite measurement was performed on AB SCIEX Qtrap1 5500 mass spectrometer (AB SCIEX, Framingham, MA, USA) ion-pairing techniques operated in negative mode as previously described [14]. A sample of 20 uL was injected on to an XSELECT HSS XP (150 × 2.1 mm × 2.5 μm) (Waters, Milford, MA, USA) column, which was equilibrated for 10 min before injecting with 100% eluent A (10 mM tributylamine, 10 mM acetic acid (pH 6.86), 5% methanol, and 2% 2-propanol). Gradient elution was set to 0% of eluent B (2-propanol) for the first 5 min, and increased to: 2% (5–9 min), 6% (9–12 min), 11% (12–13.5 min), 28% (13.5–15.5 min), and 53% (15.5–22.5 min), and returned back to 0% (22.5–23 min) and equilibrated for 10 min (23–33 min) with 100% eluent A. The flow rate was 0.4 mL min^−1^ (0–15.5 min), 0.15 mL min^−1^ (16.5–23 min), and 0.4 mL min^−1^ (27–33 min); oven temperature was set to 40 °C. The mass spectrometer was operated in multiple-reaction-monitoring (MRM) mode. The optimized parameters for 0.4-mL min^−1^ flow rate were as follows: ion-spray voltage, − 4.5 kV; curtain gas and CAD gas, 40 and 12, respectively. The capillary temperature was 500 °C.

### Data processing

HPLC and LC–MS data were processed using Chromeleon™ 7.1.3 (Thermo Scientific™) and Multi-Quant™ 3.0.2 (AB SCIEX™), respectively. For absolute quantification of intracellular metabolites, isotope ratio-based approach was used as previously described [15, 16]. This technique was performed using cell extracts grown in fully U-^13^C-labeled glucose as an internal standard for quantifying the intracellular metabolites of *P. taiwanensis* VLB120 grown on naturally labeled glucose. All statistical analyses were done using R (R Development Core Team [17]) and SIMCA (Umetrics, Umea, Sweden).

## Results and discussion

### Utilization of biomass hydrolysate sugars by *P. taiwanensis* VLB120

Hydrolysis of lignocellulosic biomass results in a mixture of sugars including the hexoses glucose, galactose, and mannose, and the pentoses xylose and arabinose [18]. In most cases, these mixtures can only be metabolized partly or sequentially, with glucose being the preferred carbon source [19–23].

**Fig. 1:**
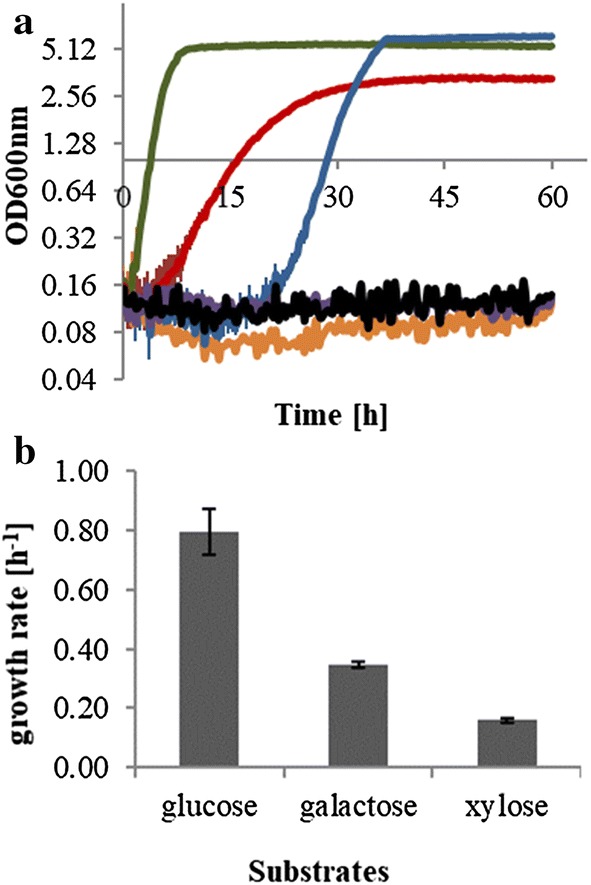
Growth profile of *P. taiwanensis* VLB120 under aerobic condition on different lignocellulosic biomass hydrolysate-derived sugars: **a** growth curve (plotted in semi-logarithm scale, *Y*-axes, log10) and **b** specific growth rate. Cells were inoculated in minimal media supplemented with 4.5 g L^−1^ of each carbon source. Glucose, green; xylose, red; mannose, orange; galactose, blue; arabinose, violet; rhamnose, black. Error bars correspond to the standard deviation of three biological replicate cultures

As shown in Fig. 1a, *P. taiwanensis* VLB120 is able to efficiently utilize glucose, xylose, and galactose despite exhibiting a prolonged lag phase in case of galactose, which lasted for up to 21 h. The strain converts glucose and xylose to their respective sugar acids, gluconate and xylonate, respectively, in the periplasmic space by glucose dehydrogenase, and the products are further transported to the cytoplasm [2]. In all cases, no quantifiable byproduct formation was detected, which indicates that the majority of carbon source is channeled to CO_2_ and biomass formation. No growth of *P. taiwanensis* VLB120 was observed when using mannose, arabinose, and rhamnose as sole carbon source.

Growth of *P. taiwanensis* VLB120 was also assessed on other carbon sources including sodium acetic acid, sodium benzoate, glycerol, and mixture of different carbon sources. The results (Additional file 1 Fig. S1) indicate that *P. taiwanensis* VLB120 was able to grow on these compounds as sole source of carbon and energy.

**Fig. 2:**
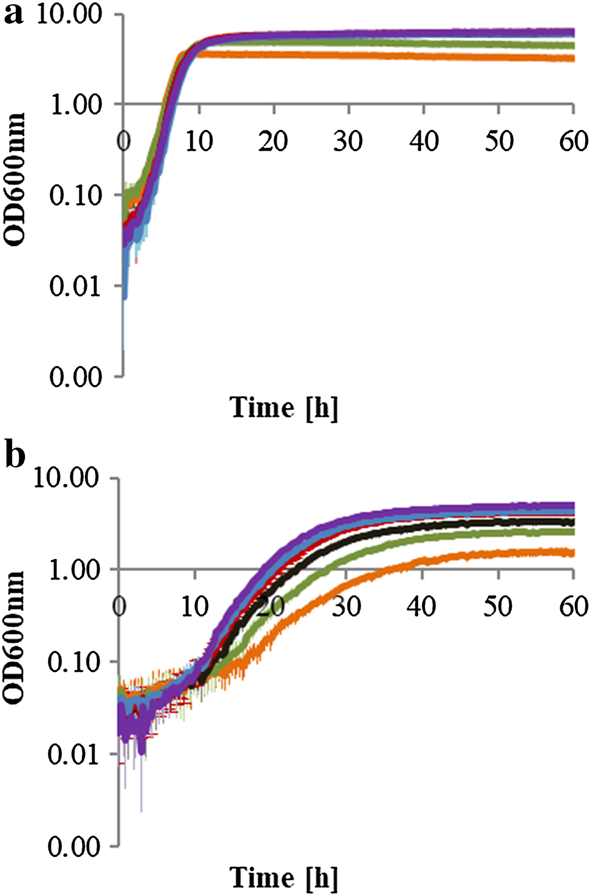
Growth curves of *P. taiwanensis* VLB120 grown under aerobic condition on minimal medium with glucose (**a**) or xylose (**b**) supplied at different concentration levels: 15 mM, orange; 25 mM, green; 35 mM, black; 45 mM, red; 55 mM, blue; 65 mM, violet. Growth curve was plotted in semi-logarithm scale (*Y*-axes, log10) from optical density (OD) measurements at 600 nm. Error bars correspond to the standard deviation of three biological replicate cultures

Since the initial concentration of sugars in biomass hydrolysates varies among different biomass sources, the effect of initial glucose and xylose concentration on *P. taiwanensis* VLB120 growth was also examined at different concentration levels, 15, 25, 35, 45, 55, and 65 mM (Fig. 2). The results indicate that the specific growth rate of *P. taiwanensis* VLB120 did not change significantly with varying initial concentrations. In contrast, the initial specific growth rate of *P. taiwanensis* VLB120 was increased with increasing xylose concentrations. This is directly related to the affinity xylose transporter which control xylose utilization. The degree to which the transporter controls the xylose uptake rate is dependent on the substrate concentration in the medium [24].

### Effect of biomass hydrolysate inhibitors on *P. taiwanensis* VLB120 growth

**Fig. 3:**
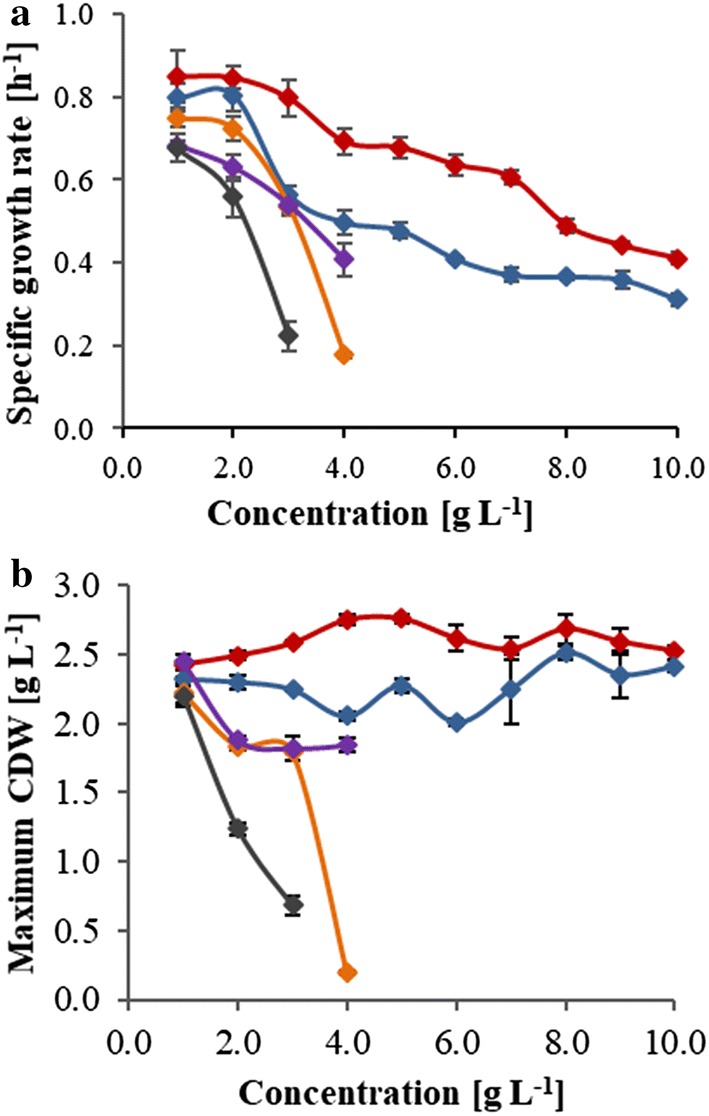
Inhibitory effects of acetic acid, formic acid, levulinic acid, vanillin, furfural, and 5-HMF on specific growth rate (**a**), and final biomass (**b**) of *P. taiwanensis* VLB120 grown on minimal medium supplemented with 4.5 g L^−1^ of glucose under aerobic condition. Acetic acid, red; formic acid, blue; vanillin, orange; furfural, violet; 5-HMF, black. Error bars correspond to the standard deviation of three biological replicates. CDW, cell dry weight

The growth inhibitory effect of acetic acid, formic acid, vanillin, furfural, and 5-HMF on growth of *P. taiwanensis* VLB120 was evaluated at different concentration levels using the Growth Profiler 960 (EnzyScreen, Heemstede, The Netherlands). The results showed that the inhibitory effect of the tested compounds varied with respect to lag phase, specific growth rate, and biomass yield compared to the control cultures grown under the same conditions without addition of inhibitors. The presence of furfural and 5-HMF in the media resulted in a prolonged lag phase and low cell density, respectively. The lag phase started to elongate from 0.98 to 24.42 h as the concentration of furfural in the fermentation broth increased from 0 to 3 g L^−1^ (Table 1). 5-HMF reduced the final cell density by 73% (Fig. 3b) at a concentration level of 3 g L^−1^. It was also observed that both furfural and 5-HMF reduced the specific growth rate (Fig. 3a) compared to the reference medium.

**Table 1:** Effects of hydrolysis-derived inhibitors on the lag phase of *P. taiwanensis* VLB120

Concentration [g L^−1^]	Lag phase (*h*)^a^					
	Acetic acid	Formic acid	Levulinic acid	Furfural	5-HMF	Vanillin
0	0.98	0.98	0.98	0.98	0.98	0.98
1	2.32	2.98	2.32	2.32	6.97	1.98
2	2.32	2.98	2.32	7.58	13.99	5.98
3	2.98	2.98	2.32	24.42	21.92	15.65
4	2.98	3.65	2.65	42.80	n/a	32.63
6	2.98	5.32	2.98	n/a	n/a	n/a
8	11.32	5.32	3.32	n/a	n/a	n/a
10	16.65	5.32	3.65	n/a	n/a	n/a

n/a: no growth in 60 h

^a^Lag phase is defined as the time needed to reach 2% of the maximum cell dry weight [25]

The effect of vanillin was comparable to that of furfural and 5-HMF. The lag phase was prolonged by 15 h, while the specific growth rate was reduced by 38% and the final biomass titer was decreased by 18% at a concentration level of 3 g L^−1^ of the corresponding inhibitory compounds. By increasing the concentration of vanillin to 4 g L^−1^, the final biomass titer was reduced by 90% and the lag phase was prolonged to 33 h (Table 1). A complete inhibition of growth of *P. taiwanensis* was observed when the concentration of 5-HMF, furfural, and vanillin exceeded 3, 4, and 4 g L^−1^, respectively (Additional file 1: Fig. S2). This might be caused by the pH drop due to the formation of the corresponding acid form of the added inhibitors.

Acetic acid and formic acid showed a similar inhibitory effect on cell growth. Both compounds slightly increased the final biomass of *P. taiwanensis* VLB120, as shown in Additional file 1: Fig. S2, but reduced the growth rate as their concentration increased (Fig. 3). The main difference of these two inhibitors was observed as the concentration of acetic acid exceeded 6 g L^−1^ where after the lag phase was clearly elongated compared to formic acid (Table 1), similar as described previously for yeast [25].

### Determination of inhibitory threshold concentrations affecting *P. taiwanensis* VLB120 growth

**Fig. 4:**
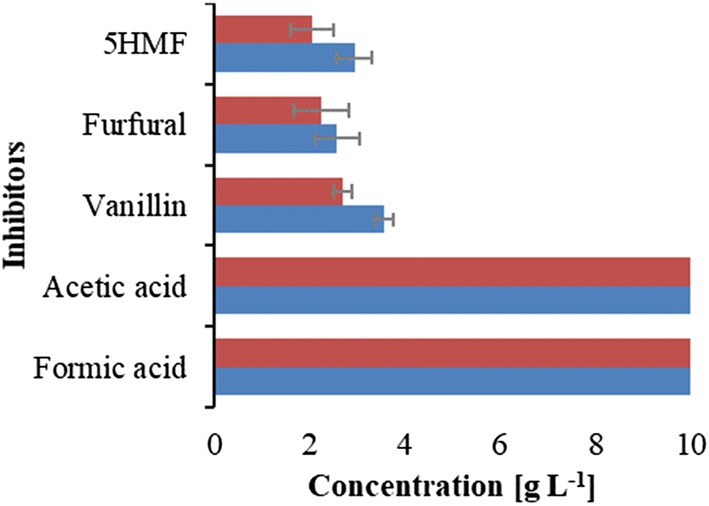
IC50 (red) and IC90 (blue) values of lignocellulose-derived inhibitors for *P. taiwanensis* VLB120 after 24 h of cultivation. Abbreviations: IC50 and IC90 indicate inhibitory concentrations that reduce the growth of *P. taiwanensis* VLB120 with 50 and 90%, respectively. Error bars indicate standard deviations of three independent cultures

The inhibitory threshold concentration values of acetic acid, formic acid, furfural, 5-HMF, and vanillin that reduced the growth of *P. taiwanensis* VLB120 by 50% and 90% (IC50 and IC90) were estimated after 24 h of cultivation (Fig. 4). The IC50 and IC90 values of each of the inhibitory compounds were calculated by generating an inhibition curve for each inhibitor. This approach has frequently been applied as a general toxicity indicator for potential inhibitors [26, 27].

The concentrations resulting in a 50% reduction of *P. taiwanensis* VLB120 growth (IC50) with 5-HMF and furfural are highly comparable with the reported values for *Thermoanaerobacter pseudethanolicus* 39E [28], *Bacillus coagulans* MXL-9 [29], *S. cerevisiae* CBS1200 [30], and *Zymomonas* *mobilis* ATCC 10988 [30]. Based on IC50 values, 5-HMF provided the strongest inhibition followed by furfural and vanillin, respectively. These results are in line with the previous reports which confirmed that furfural and 5-HMF were identified as main inhibitors in biomass hydrolysates [26, 31–33]. In contrast, IC50 values for acetic acid and formic acid were above the highest tested concentration (10 g L^−1^), which means that this concentration was not high enough to reduce the growth of *P. taiwanensis* VLB120 by 50%. These values are higher compared to well-known production strains such as *E. coli* (IC50 2.5-g L^−1^ formic acid and 9.0-g L^−1^ acetic acid) [34]. This indicates that *P. taiwanensis* VLB120 is highly tolerant to acetic acid and formic acid when glucose is used as a sole carbon source.

### Degradation capacity of lignocellulosic biomass-derived inhibitors by *P. taiwanensis* VLB 120

A number of microorganisms have evolved different strategies including reduction and oxidation processes to detoxify inhibitory compounds [35–38]. For instance, *Gluconacetobacter xylinus* oxidizes furfural and 5-HMF directly to furoic acid and 5-hydroxymethyl-2-furoic acid, respectively [39]. Microorganisms such as *E. coli* and *S. cerevisiae* not possessing oxidative degradation pathways for furan aldehydes [19] use their native oxidoreductases to reduce furan aldehydes to furan alcohols under anaerobic conditions [34, 40].

In this study, the metabolic response and degradation potential of lignocellulosic biomass-derived inhibitory compounds by *P. taiwanensis* VLB 120 was investigated using a targeted metabolomics approach. Since some of these inhibitors have structural similarity and share the same degradation pathway, only acetic acid, levulinic acid, furfural, and vanillin were considered for the metabolomics study. For a reliable quantitative metabolomics analysis, 2 g L^−1^ of each inhibitory compound was chosen based on half maximal inhibitor concentration (IC50) value assuming that this concentration level is sufficiently high to affect cell behavior and metabolism without being lethal.

**Fig. 5:**
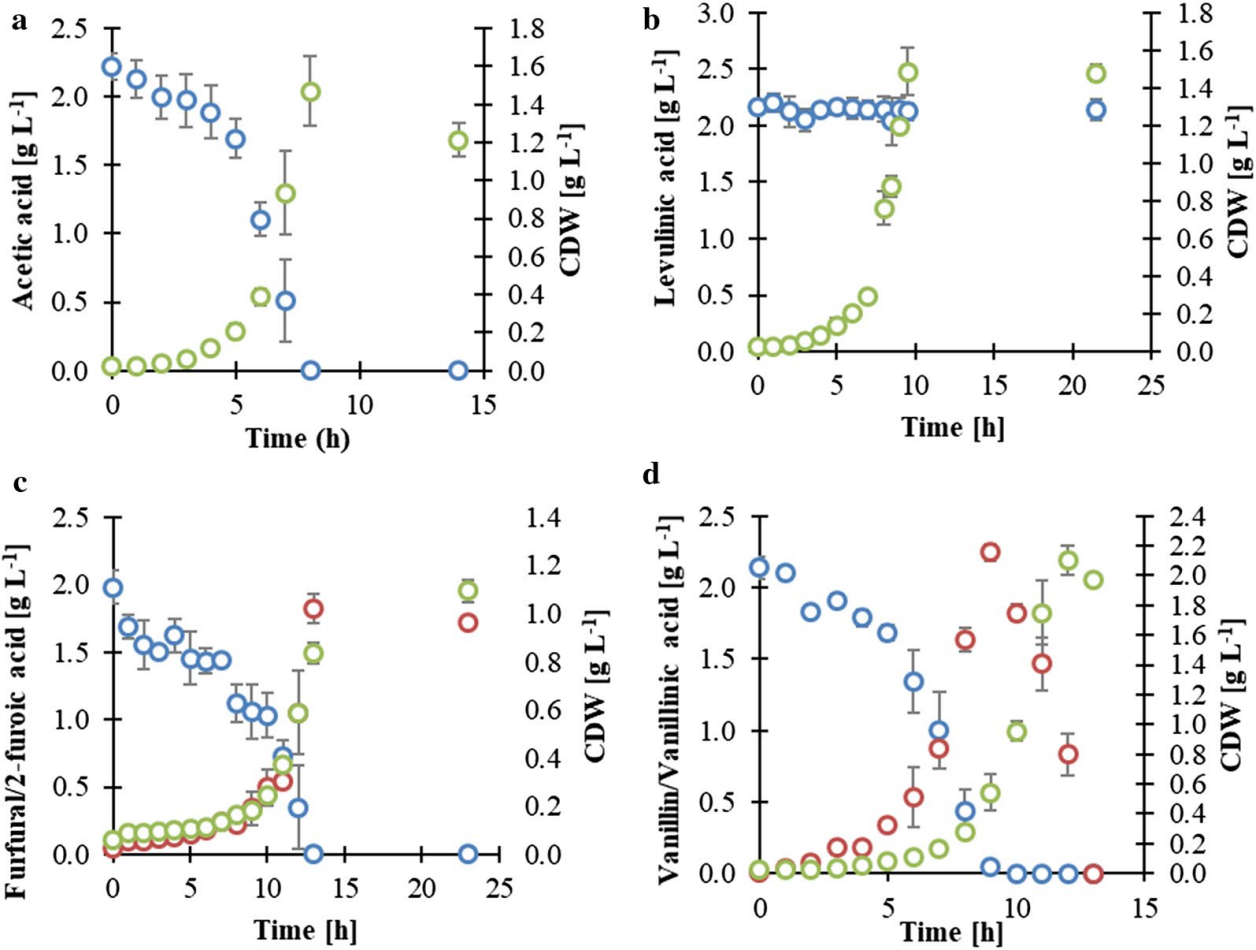
Conversion capacity of acetic acid (**a**), levulinic acid (**b**), furfural (**c**), and vanillin (**d**) by *P. taiwanensis* VLB120 grown on minimal medium supplemented with 4.5 g L^−1^ of glucose at stirrer speed of 800 rpm, temperature 30 °C and pH 7. Blue represents acetic acid (**a**), levulinic acid (**b**), furfural (**c**), and vanillin (**d**); red represents furoic acid (**c**) and vanillic acid (**d**); green represents cell dry weight (CDW). Error bars indicate standard deviations of three independent cultures

As shown in Fig. 5, the concentration of acetic acid, formic acid, furfural, 5-HMF, and vanillin was decreased during the cultivation process, suggesting their conversion or consumption, while the concentration of levulinic acid remained constant throughout the cultivation.

Based on the supernatant analysis, *P. taiwanensis* VLB120 is able to oxidize vanillin and furfural to vanillic acid and 2-furoic acid, respectively (Fig. 5c, d). Vanillic acid was further metabolized to protocatechuic acid and eventually entered the central carbon pathway via the β-ketoadipate route [41, 42], whereas 2-furoic acid was secreted outside the cells as the conversion of furfural to 2-furoic acid carried out on the outer surface of the cells [43] and remained in the fermentation broth without further conversion.

There was no significant growth of cells observed until the majority of furfural and vanillin in the medium were converted to their corresponding acid, which would also explain the long lag phase. This indicates that the presence of these inhibitors in the media obstructed the growth of *P. taiwanensis* VLB120. However, their corresponding acids had a less toxic effect and, therefore, allowed growth of *P. taiwanensis* VLB120. These findings are in agreement with the previous studies that proved the aldehyde form as the most toxic one of several aromatic inhibitory compounds, whereas the corresponding acids were less toxic, while the alcohol form was the least toxic one [25, 44–46].

Acetic acid was completely consumed from the fermentation broth after 8 h of cultivation (Fig. 5a). This indicates that acetate was activated to acetyl-CoA and completely metabolized from the fermentation broth via the TCA cycle to carbon dioxide, which agrees with findings of Matano et al. [47] and Gebhardt et al. [48].

Furthermore, vanillin and furfural appeared to cause a pronounced stress response, resulting in a substantial reduction in specific glucose uptake and specific growth rate during the oxidation process. In contrast, when cultivating *P. taiwanensis* VLB120 under acetic acid and levulinic acid conditions, the specific glucose uptake rate was increased approx. by 40 and 9% (Table 2), respectively, compared to the control condition. The decreased specific growth rates and increased specific glucose uptake rate by *P. taiwanensis* VLB120 reflect the additional energy required either to pump out the inhibitory compound from the cell or to transport proton through the plasma membrane to adjust the intracellular pH to a threshold at which essential enzymes can function [49, 50].

**Table 2:** Physiological parameters of *P. taiwanensis* VLB120 during growth on glucose in the presence of inhibitory compounds

Physiological parameters	Unit	Acetic acid	Levulinic acid	Furfural	Vanillin	Control
Specific growth rate	h^−1^	0.58 ± 0.02	0.45 ± 0.06	0.19 ± 0.01	0.33 ± 0.04	0.69 ± 0.03
Specific glucose uptake	g g^−1^CDW h^−1^	10.45 ± 0.58	8.13 ± 1.69	1.72 ± 0.12	4.25 ± 1.70	7.44 ± 0.42
Specific gluconate production rate	g g^−1^CDW h^−1^	10.70 ± 0.39	7.72 ± 1.72	1.49 ± 0.10	3.74 ± 1.52	6.04 ± 0.88
Biomass yield on glucose	g g^−1^CDW	0.05 ± 0.00	0.06 ± 0.00	0.10 ± 0.00	0.12 ± 0.02	0.09 ± 0.01

Since *P. taiwanensis* VLB120 exhibits biphasic growth (glucose and gluconate phases), only glucose phase was considered for the determination of specific growth rate, specific glucose uptake/gluconate production rate, and biomass yield (Additional file 1: Fig. S3)

*Pseudomonas taiwanensis* VLB120 completely metabolized glucose to gluconate in the pretense of acetic acid. This could be related to the direct utilization of acetic acid as an additional carbon and energy source.

### Effect of inhibitory compounds on the *P. taiwanensis* VLB120 metabolome composition

Comparative analyses of the primary and key intermediate metabolites were considered to investigate the metabolic response of *P. taiwanensis* VLB120 to lignocellulose-derived inhibitors. For each tested inhibitor, intracellular metabolites were extracted with boiling ethanol from exponentially growing *P. taiwanensis* VLB120 cultures at an optical density (OD_600 nm_) of 0.4–0.6.

In total, 80 metabolites from different classes, including sugars phosphates, amino acids, organic acids, redox cofactors, nucleosides/bases, and nucleotides, were quantified across all conditions. These metabolites do not cover the entire metabolome of *P. taiwanensis* VLB120; however, they possess an essential role in central metabolism. To provide comparative information regarding the metabolic differences among each group, a principal component analysis (PCA) was performed.

**Fig. 6:**
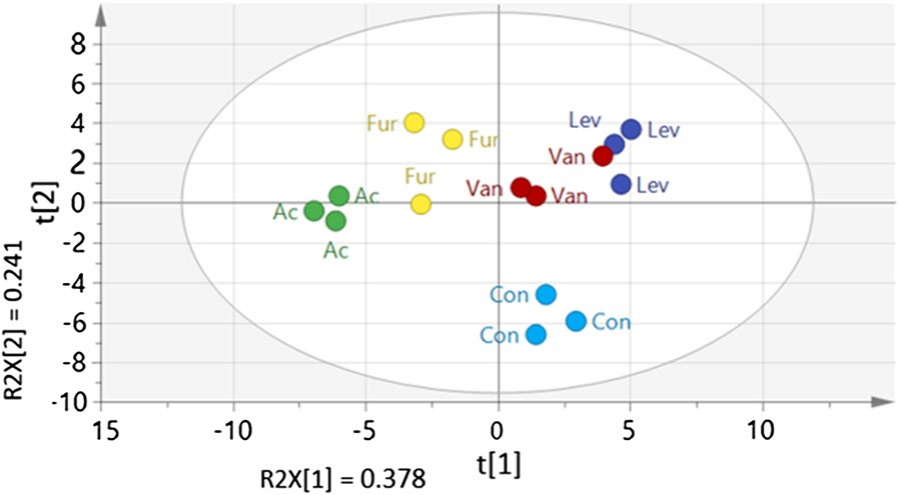
Principal component analysis (PCA) score plots of metabolic profiles in *P. taiwanensis* VLB120 under the treatment of multiple inhibitors. *Fur* furfural, *Van* vanillin, *Lev* levulinic acid, *Ac* acetic acid, *Con* control

Approximately, 62% of the total variance in the data was represented by the first two principal components (Fig. 6). Samples from different treatments separated clearly from control sample, indicating an adjustment of intracellular metabolism of the *P. taiwanensis* strain in response to inhibitors. Metabolites including nucleotides, redox cofactors, and sugar phosphates particularly contributed to separate the groups (Additional file 1: Fig. S5). Samples treated with vanillin, furfural, and levulinic acid were moved to the upper part of PC(1), indicating relative similarity of their effect on the concentration of several metabolites including, ATP, ADP, and NADPH. This is corroborated by heat map cluster analysis based on the degree of similarity of metabolite abundance profiles (Additional file 1: Fig. S6). Levulinic acid and vanillin-treated samples were positioned close to each other, indicating that these compounds effected the intracellular metabolome composition in a similar way.

Nucleotide monophosphates (e.g., AMP, CMP, IMP, GMP, and UMP) seemed to have high influence in separating the samples treated with acetic acid from the rest of the groups. This could be related to the requirement of ATP to convert acetate to acetyl-CoA which results in the production of AMP. The observed low intracellular concentration of acetyl-CoA was mainly related to its utilization for re-generation of ATP via the TCA cycle [51, 52].

**Fig. 7:**
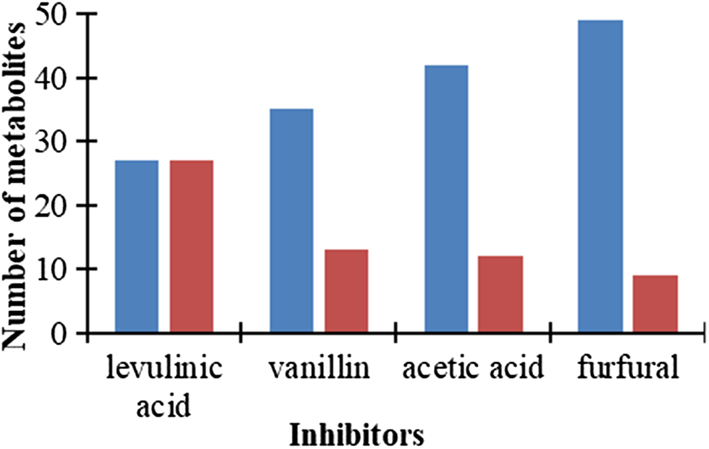
Total number of metabolites that exhibited more than 20% change in abundance compared to the control samples. Lower abundant, blue; higher abundant, red

As shown in Fig. 7, the number of metabolites that showed significantly increased or decreased levels during cultivation with levulinic acid was identical. However, the majority of quantified metabolites exhibited lower concentrations compared to the control samples in the presence of furfural, acetic acid, and vanillin (Fig. 7). The concentration of some of the metabolites was decreased more than twofold in the presence of acetic acid (e.g., fructose 1,6-bisphosphate, 6-phospho gluconate, and acetyl-CoA), furfural (e.g., adenine, inosine, and oxidized glutathione), levulinic acid (e.g., AMP, IMP, and adenine), and vanillin (e.g., AMP and UDP glucuronate) in comparison to the control samples. This indicates that there was no unique pattern of metabolic rearrangement in *P. taiwanensis* VLB120 to cope with the exposure to inhibitory compounds.

The mechanisms that lead to the observed change of intracellular concentrations of other classes of metabolite (e.g., sugar phosphates, organic acids, and amino acids) could be the consequence of changes in cellular energetics and redox state of the cell. Metabolites including ATP and NADPH are generally reported to have key functions in the survival of any organism in a stressful environment [53, 54]. This is due to the fact that microorganisms require both NADPH-dependent detoxification and ATP-dependent efflux to cope with inhibitors [55]. Since those metabolites are a fundamental requirement for the maintenance of metabolism, energy generation, and growth, their perturbations may induce widespread changes in metabolism [53, 54, 56–62].

To investigate the role of cellular energetics and redox carrier metabolites in *P. taiwanensis* VLB120 during growth with inhibitory compounds, the level of ATP and NADPH/NADP ratio was determined in both control sample and samples treated with inhibitors. Since several enzymes are regulated by the ratio between reduced and oxidized cofactors [53], NADPH/NADP ratio was considered in steady of absolute concentration of NADPH.

At the time of sampling, the oxidation of vanillin and furfural to their corresponding acids were ongoing, while acetic acid was metabolizing. The concentration of levulinic acid remained constant throughout the fermentation process.

**Fig. 8:**
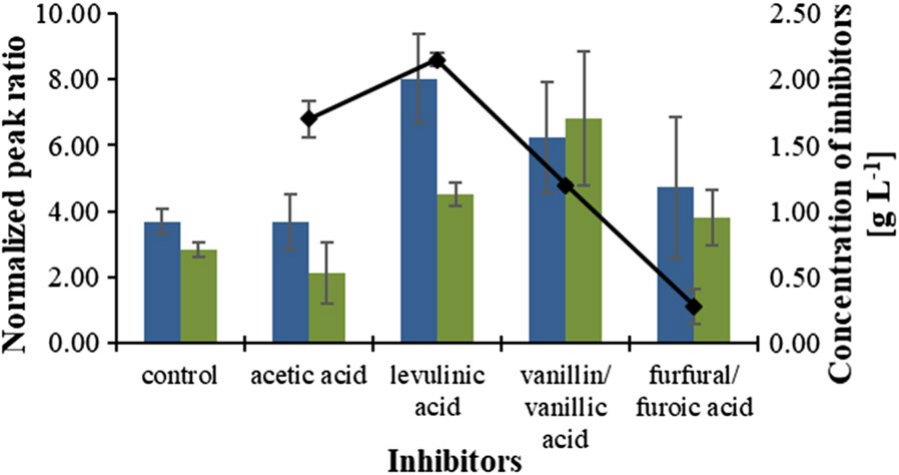
Effect of inhibitors on energy state and redox carrier of glucose-utilizing *P. taiwanensis* VLB120. The bars indicate the peak ratio of ATP (blue) and NADPH/NADP (green); the black line represents the concentration of acetic acid, levulinic acid, vanillic acid, and 2-furoic acid at the time of sampling. Peak ratio is the height ratio between the U ^13^C and ^12^C metabolites normalized to biomass. Error bars indicate standard deviations of three independent cultures

As indicated in Fig. 8, the levels of ATP and NADPH/NADP ratio (which was directly correlated to the absolute concentration of NADPH) were markedly increased in the cells treated with levulinic acid, vanillin, and furfural compared to that of the control. This enhanced level of ATP could be related to the bacterial cells generating more ATP to pump out the inhibitors from the cell that enter the cytoplasm through passive diffusion. The extent to which a compound can enter in the cell cytoplasm depends on their hydrophobicity potentials (log *P*). A high value of log P indicates that the compound can readily translocate into the cell across cell membrane [63]. The log *P* value of levulinic acid is relatively high (1.34) followed by vanillic acid (1.20), 2-furoic acid (0.73), and acetic acid (− 0.32) [63]. This indicates that levulinic acid can easily enter to the cytoplasm, and therefore, the cells treated with this inhibitory compound are required to generate more ATP than the cell treated with other inhibitors to export it out.

Similarly, the increased level of NADPH/NADP ratio provided reducing power to deal with the oxidative stress that caused by the inhibitors [64]. This observation is in agreement with a previous study, showing that *Pseudomonas fluorescens* produced high NADPH to cope with oxidative stress [62]. NADPH diminishes oxidative stress and provides the reductive environment necessary for cellular activities [62]. For instance, the production of ATP via oxidative phosphorylation cannot be effective for aerobic organism growing under stress conditions unless it is equipped with enough supply of NADPH that provides a reductive environment [57, 58, 62]. In the presence of acetic acid, NADPH/NADP ratio was slightly impaired, while the concentration of ATP was unchanged, which was also in reasonable agreement with a previous study [65]. The observed minimal effect of acetic acid on that metabolite could be related to the direct utilization of acetate by *P. taiwanensis* as an additional carbon and energy source.

Overall, there appeared to be a metabolic shift in *P. taiwanensis* to enhance the levels of ATP and NADPH/NADP ratio to cope with the stress imposed by inhibitors. Thus, adequate supply of these metabolites is essential for the survival and reproduction of *P. taiwanensis* in the presence of biomass-derived inhibitors.

## Conclusions

In this study, the tolerance and metabolic responses of *P. taiwanensis* VLB120 to biomass hydrolysate-derived inhibitors were investigated. The overall results suggest that the tested inhibitors affect *P. taiwanensis* VLB120 physiology in various ways with respect to lag phase, specific growth rate, and biomass yield. To overcome these effects, *P*. *taiwanensis* VLB120 adopt different resistance mechanisms, including detoxification, efflux, and repair, which require additional cellular energy and resources. *P. taiwanensis* VLB120 went through metabolic rearrangement to generate more ATP and NADPH to mitigate the stress imposed by inhibitors.

In general, efficiently use of biomass hydrolysate as fermentation media requires microorganism that can utilize both C6 and C5 sugars and able to tolerate the inhibitory compounds formed during biomass pretreatment process. *P. taiwanensis* VLB120 showed high tolerance towards biomass hydrolysate-derived inhibitors and efficiently utilize glucose, xylose, and galactose as a carbon and energy source. This indicates that the physiology of *P. taiwanensis* VLB120 matches the aforementioned basic requirements for growth on biomass hydrolysate.

## Additional file

**Additional file 1: Fig. S1.** Evaluation of different carbon sources for growth by *P. taiwanensis* VLB120. **Fig. S2.** Effect of biomass hydrolysate derived inhibitors on *P. taiwanensis* VLB120. **Fig. S3.** Impact of inhibitory compounds on specific glucose uptake rate. **Fig. S4.** Venn diagram. **Fig. S5.** PCA loading plot. **Fig. S6.** Heat map based on intracellular metabolites data.

## References

[1] Volmer J, Neumann C, Bühler B, Schmid A (2014). Engineering of *Pseudomonas taiwanensis* VLB120 for constitutive solvent tolerance and increased specific styrene epoxidation activity. Appl Environ Microbiol.

[2] Köhler KAK, Blank LM, Frick O, Schmid A (2015). d-Xylose assimilation via the Weimberg pathway by solvent-tolerant *Pseudomonas taiwanensis* VLB120. Environ Microbiol.

[3] Köhler KAK, Rückert C, Schatschneider S, Vorhölter FJ, Szczepanowski R, Blank LM (2013). Complete genome sequence of *Pseudomonas* sp. strain VLB120 a solvent tolerant, styrene degrading bacterium, isolated from forest soil. J Biotechnol.

[4] Hong SJ, Park GS, Khan AR, Jung BK, Shin JH (2017). Draft genome sequence of a caprolactam degrader bacterium: *Pseudomonas taiwanensis* strain SJ9. Braz J Microbiol..

[5] Thomashow LS (1997). Frequency of antibiotic-producing *Pseudomonas* spp. in natural environments. Appl Environ Microbiol..

[6] Dowling DN, O’Gara F (1994). Metabolites of *Pseudomonas* involved in the biocontrol of plant disease. Trends Biotechnol.

[7] Kulkarni RS, Kanekar PP (1993). Bioremediation of €-caprolactam from nylon-6 waste water by use of *Pseudomonas aeruginosa* MCM B-407. Curr Microbiol.

[8] Roselló-Mora R, Lalucat J, García-Valdés E (1994). Comparative biochemical and genetic analysis of napthalene degradation among *Pseudomonas* stutzeri strains. Appl Environ Microbiol.

[9] Nielsen DR, Leonard E, Yoon SH, Tseng HC, Yuan C, Prather KLJ (2009). Engineering alternative butanol production platforms in heterologous bacteria. Metab Eng.

[10] Mills TY, Sandoval NR, Gill RT (2009). Cellulosic hydrolysate toxicity and tolerance mechanisms in *Escherichia coli*. Biotechnol Biofuels.

[11] Jansson LJ, Martin C (2016). Pretreatment of lignocellulose: formation of inhibitory by-products and strategies for minimizing their effects. Bioresour Technol.

[12] Hartmans S, Smits JP, Van der Werf MJ, Volkering F, De Bont JAM (1989). Metabolism of styrene oxide and 2-phenylethanol in the styrene-degrading *Xanthobacter* strain 124X. Appl Environ Microbiol.

[13] Wordofa GG, Kristensen M, Schrübbers L, McCloskey D, Forster J, Schneider K (2017). Quantifying the metabolome of *Pseudomonas taiwanensis* VLB120: evaluation of hot and cold combined quenching/extraction approaches. Anal Chem..

[14] McCloskey D, Utrilla J, Naviaux RK, Palsson BO, Feist AM (2014). Fast Swinnex filtration (FSF): a fast and robust sampling and extraction method suitable for metabolomics analysis of cultures grown in complex media. Metabolomics..

[15] Wu L, Mashego MR, Van Dam JC, Proell AM, Vinke JL, Ras C (2005). Quantitative analysis of the microbial metabolome by isotope dilution mass spectrometry using uniformly 13C-labeled cell extracts as internal standards. Anal Biochem.

[16] Mashego MR, Wu L, Van Dam JC, Ras C, Vinke JL, Van Winden WA (2004). MIRACLE: mass isotopomer ratio analysis of U-13C-labeled extracts. a new method for accurate quantification of changes in concentrations of intracellular metabolites. Biotechnol Bioeng.

[17] R Development Core Team R. R: A language and environment for statistical computing. 2011. 10.1007/978-3-540-74686-7.

[18] Zaldivar J, Nielsen J, Olsson L (2001). Fuel ethanol production from lignocellulose: a challenge for metabolic engineering and process integration. Appl Microbiol Biotechnol.

[19] Nieves LM, Panyon LA, Wang X (2015). Engineering sugar utilization and microbial tolerance toward lignocellulose conversion. Front Bioeng Biotechnol..

[20] Dien BS, Iten L, Bothast RJ (1999). Conversion of corn fiber to ethanol by recombinant *E. coli* strain FBR3. J Ind Microbiol Biotechnol.

[21] Han JH, Park JY, Yoo KS, Kang HW, Choi GW, Chung BW (2011). Effect of glucose on xylose utilization in *Saccharomyces cerevisiae* harboring the xylose reductase gene. Arch Microbiol.

[22] Yanase H, Miyawaki H, Sakurai M, Kawakami A, Matsumoto M, Haga K (2012). Ethanol production from wood hydrolysate using genetically engineered *Zymomonas mobilis*. Appl Microbiol Biotechnol.

[23] Xia T, Eiteman MA, Altman E (2012). Simultaneous utilization of glucose, xylose and arabinose in the presence of acetate by a consortium of *Escherichia coli* strains. Microb Cell Fact.

[24] Runquist D, Hahn-Hägerdal B, Rådström P (2010). Comparison of heterologous xylose transporters in recombinant *Saccharomyces cerevisiae*. Biotechnol Biofuels.

[25] Zha Y, Muilwijk B, Coulier L (2012). Inhibitory compounds in lignocellulosic biomass hydrolysates during hydrolysate fermentation processes. J Bioprocess Biotech..

[26] Wang W, Yang S, Hunsinger GB, Pienkos PT, Johnson DK (2014). Connecting lignin-degradation pathway with pre-treatment inhibitor sensitivity of *Cupriavidus necator*. Front Microbiol..

[27] Franden MA, Pilath HM, Mohagheghi A, Pienkos PT, Zhang M (2013). Inhibition of growth of *Zymomonas mobilis* by model compounds found in lignocellulosic hydrolysates. Biotechnol Biofuels.

[28] Clarkson SM, Hamilton-Brehm SD, Giannone RJ, Engle NL, Tschaplinski TJ, Hettich RL (2014). A comparative multidimensional LC–MS proteomic analysis reveals mechanisms for furan aldehyde detoxification in *Thermoanaerobacter pseudethanolicus* 39E. Biotechnol Biofuels.

[29] Bischoff KM, Liu S, Hughes SR, Rich JO (2010). Fermentation of corn fiber hydrolysate to lactic acid by the moderate *thermophile Bacillus coagulans*. Biotechnol Lett.

[30] Delgenes JP, Moletta R, Navarro JM (1996). Effects of lignocellulose degradation products on ethanol fermentations of glucose and xylose by *Saccharomyces cerevisiae*, *Zymomonas mobilis*, *Pichia stipitis*, and *Candida shehatae*. Enzyme Microb Technol.

[31] Klinke HB, Thomsen AB, Ahring BK (2004). Inhibition of ethanol-producing yeast and bacteria by degradation products produced during pre-treatment of biomass. Appl Microbiol Biotechnol.

[32] Wierckx N, Koopman F, Bandounas L, De Winde JH, Ruijssenaars HJ (2010). Isolation and characterization of *Cupriavidus basilensis* HMF14 for biological removal of inhibitors from lignocellulosic hydrolysatembt. Microb Biotechnol.

[33] Palmqvist E, Hahn-Hägerdal B (2000). Fermentation of lignocellulosic hydrolysates. I: inhibition and detoxification. Bioresour Technol.

[34] Zaldivar J, Martinez A, Ingram LO (1999). Effect of selected aldehydes on the growth and fermentation of ethanologenic *Escherichia coli*. Biotechnol Bioeng.

[35] Booth IR, Ferguson GP, Miller S, Li C, Gunasekera B, Kinghorn S (2003). Bacterial production of methylglyoxal: a survival strategy or death by misadventure?. Biochem Soc Trans.

[36] Herring CD, Blattner FR (2004). Global transcriptional effects of a suppressor tRNA and the inactivation of the regulator frmR. J Bacteriol.

[37] Marx CJ, Miller JA, Lidstrom ME, Chistoserdova L (2004). Multiple formaldehyde oxidation/detoxification pathways in *Burkholderia fungorum* LB400 multiple formaldehyde oxidation/detoxification pathways in *Burkholderia fungorum* LB400. J Bacteriol.

[38] Jarboe LR (2011). YqhD: a broad-substrate range aldehyde reductase with various applications in production of biorenewable fuels and chemicals. Appl Microbiol Biotechnol.

[39] Zhang S, Winestrand S, Chen L, Li D, Jonsson LJ, Hong F (2014). Tolerance of the nanocellulose-producing bacterium *gluconacetobacter xylinus* to lignocellulose-derived acids and aldehydes. J Agric Food Chem.

[40] Zaldivar J, Martinez A, Ingram LO (2000). Effect of alcohol compounds found in hemicellulose hydrolysate on the growth and fermentation of ethanologenic *Escherichia coli*. Biotechnol Bioeng.

[41] Mohan K, Phale P (2017). Carbon source-dependent inducible metabolism of veratryl alcohol and ferulic acid in *Pseudomonas putida* CSV86. Appl Environ Microbiol.

[42] Álvarez-Rodríguez ML, Belloch C, Villa M, Uruburu F, Larriba G, Coque JJR (2003). Degradation of vanillic acid and production of guaiacol by microorganisms isolated from cork samples. FEMS Microbiol Lett.

[43] Eilers FI, Sussman AS (1970). Conversion of furfural to furoic acid and furfuryl alcohol by *Neurospora ascospores*. Planta.

[44] Klinke HB, Olsson L, Thomsen AB, Ahring BK (2003). Potential inhibitors from wet oxidation of wheat straw and their effect on ethanol production of *Saccharomyces cerevisiae*: wet oxidation and fermentation by yeast. Biotechnol Bioeng.

[45] Hahn-Hägerdal B (1998). Detoxification of wood hydrolysate with laccase and peroxidase from the white-rot fungus *T. versicolor*. Appl Microb Biotechnol..

[46] Larsson S, Quintana-Sáinz A, Reimann A, Nilvebrant NO, Jönsson LJ (2000). Influence of lignocellulose-derived aromatic compounds on oxygen-limited growth and ethanolic fermentation by *Saccharomyces cerevisiae*. Appl Biochem Biotechnol.

[47] Matano C, Meiswinkel TM, Wendisch VF, Watson RR, Preedy VR, Zibadi S (2014). Amino acid production from rice straw hydrolyzates. Wheat and rice in disease prevention and health.

[48] Gebhardt NA, Thauer RK, Linder D, Kaulfers PM, Pfennig N (1985). Mechanism of acetate oxidation to CO_2_ with elemental sulfur in *Desulfuromonas acetoxidans*. Arch Microbiol.

[49] Suko AV, Bura R (2016). Enhanced xylitol and ethanol yields by fermentation inhibitors in steam-pretreated lignocellulosic biomass. Ind Biotechnol..

[50] Guo Z, Olsson L (2014). Physiological response of *Saccharomyces cerevisiae* to weak acids present in lignocellulosic hydrolysate. FEMS Yeast Res.

[51] Bräsen C, Schönheit P (2004). Regulation of acetate and acetyl-CoA converting enzymes during growth on acetate and/or glucose in the halophilic archaeon *Haloarcula marismortui*. FEMS Microbiol Lett.

[52] Liang MH, Qv XY, Jin HH, Jiang JG (2016). Characterization and expression of AMP-forming Acetyl-CoA Synthetase from *Dunaliella tertiolecta* and its response to nitrogen starvation stress. Sci Rep..

[53] Ask M, Bettiga M, Mapelli V, Olsson L (2013). The influence of HMF and furfural on redox-balance and energy-state of xylose-utilizing *Saccharomyces cerevisiae*. Biotechnol Biofuels.

[54] Messaoudi N, Gautier V, Dairou J, Mihoub M, Lelandais G, Bouloc P (2015). Fermentation and alternative respiration compensate for NADH dehydrogenase deficiency in a prokaryotic model of DJ-1-associated Parkinsonism. Microbiology.

[55] Piotrowski JS, Zhang Y, Bates DM, Keating DH, Sato TK, Ong IM (2014). Death by a thousand cuts: the challenges and diverse landscape of lignocellulosic hydrolysate inhibitors. Front Microbiol..

[56] Bloem A, Sanchez I, Dequin S, Camarasa C (2016). Metabolic impact of redox cofactor perturbations on the formation of aroma compounds in *Saccharomyces cerevisiae*. Appl Environ Microbiol.

[57] Jo SH, Son MK, Koh HJ, Lee SM, Song IH, Kim YO (2001). Control of mitochondrial redox balance and cellular defense against oxidative damage by mitochondrial NADP+ -dependent isocitrate dehydrogenase. J Biol Chem.

[58] Rydström J (2006). Mitochondrial transhydrogenase—a key enzyme in insulin secretion and potentially, diabetes. Trends Biochem Sci.

[59] Chai MF, Chen QJ, An R, Chen YM, Chen J, Wang XC (2005). NADK2, an *Arabidopsis* chloroplastic NAD kinase, plays a vital role in both chlorophyll synthesis and chloroplast protection. Plant Mol Biol.

[60] Grose JH, Joss L, Velick SF, Roth JR (2006). Evidence that feedback inhibition of NAD kinase controls responses to oxidative stress. Proc Natl Acad Sci USA..

[61] Sakuraba H, Kawakami R, Ohshima T (2005). First archaeal inorganic polyphosphate/ATP-dependent NAD kinase, from hyperthermophilic archaeon *Pyrococcus horikoshii*: cloning. Express Charact..

[62] Singh R, Mailloux RJ, Puiseux-Dao S, Appanna VD (2007). Oxidative stress evokes a metabolic adaptation that favors increased NADPH synthesis and decreased NADH production in *Pseudomonas fluorescens*. J Bacteriol.

[63] Ibraheem O, Ndimba BK (2013). Molecular adaptation mechanisms employed by ethanologenic bacteria in response to lignocellulose-derived inhibitory compounds. Int J Biol Sci..

[64] Hara M, Matsuura T, Kojima S (2015). Innovative medicine.

[65] Guo W, Chen Y, Wei N, Feng X (2016). Investigate the metabolic reprogramming of *Saccharomyces cerevisiae* for enhanced resistance to mixed fermentation inhibitors via 13C metabolic flux analysis. PLoS ONE.

