# Supplementary material for: Tolerance and metabolic response of Pseudomonas taiwanensis VLB120 towards biomass hydrolysate-derived inhibitors
Source: Biotechnol Biofuels. 2018 Jul 19;11:199. doi: 10.1186/s13068-018-1192-y (PMC6052574; doi:10.1186/s13068-018-1192-y)
Supplement: Supplementary file 1 — Additional file 1: Fig. S1. Evaluation of different carbon sources for growth by P. taiwanensis VLB120. Fig. S2. Effect of biomass hydrolysate derived inhibitors on P. taiwanensis VLB120. Fig. S3. Impact of inhibitory compounds on specific glucose uptake rate. Fig. S4. Venn diagram. Fig. S5. PCA loading plot. Fig. S6. Heat map based on intracellular metabolites data. [file 13068_2018_1192_MOESM1_ESM.docx]

Additional Information for Publication

**Tolerance and Metabolic Response of *Pseudomonas taiwanensis* VLB120 towards Biomass Hydrolysate Derived Inhibitors**

Gossa G. Wordofa^†^ and Mette Kristensen^†*^

† Novo Nordisk Foundation Center for Biosustainability, Technical University of Denmark, DK-2800 Lyngby, Denmark

Corresponding Author

*Phone: +45 24649328 Email: [metk@biosustain.dtu.dk](mailto:metk@biosustain.dtu.dk)

1. **Evaluation of different carbon sources for growth by *P. taiwanensis* VLB120**

Additional file 1: Figure S1. Growth profile of *P. taiwanensis* VLB120 in different carbon source (A) and mixed carbon sources (B). Growth rate (C) was calculated from growth curves. Aerobic cultivations were carried out in 24-well clear bottom microplate (EnzyScreen, Heemstede, The Netherlands) working volume 750 µL at 30 °C, 225 rpm. Error bars are the standard deviation of three biological replicate cultures. Abbreviations: glucose (glc), sodium benzoate (SB), sodium acetate (SA), galactose (gal), glycerol (gly) and xylose (xyl).

1. **Effect of biomass hydrolysate derived inhibitors on *P. taiwanensis* VLB120**

Additional file 1: Figure S2. Growth curve of *P. taiwanensis* VLB120 in the presence of furfural, 5-HMF, levulinic acid, formic acid, acetic acid and vanillin. Aerobic cultivations were carried out in 24-well clear bottom microplate (EnzyScreen, Heemstede, The Netherlands) working volume 750 µL at 30 °C, 225 rpm. Concentration: [g L^-1^]. Error bars are the standard deviation of three biological replicate cultures.

1. **Impact of inhibitory compounds on specific glucose uptake rate**

I II

Additional file 1: Figure S3. Growth of *P. taiwanensis* VLB120 in minimal media supplemented with 4 g L^-1^ of glucose in the presence of 2 g L^-1^ acetic acid (A), levulinic acid (B), furfural (C), or vanillin (D) and in the absence of inhibitory compound (E). The experiments were performed in 1.3-L bioreactors (SARTORIOUS ®) with 0.5 L working volume. The temperature, stirrer speed and pH were set at 30 °C, 800 rpm and 7.0, respectively. Cultures were supplied with air at a flow rate of 1 slpm, and minimum dissolved oxygen saturation level was 40%. Error bars indicate standard deviations of three independent cultures. CDW, cell dry weight. (I) glucose phase, (II) gluconate phase.

1. **Venn diagram**


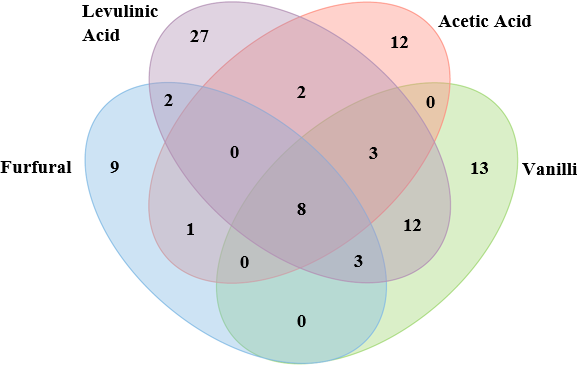


Additional file 1: Figure S4. Venn diagram illustrating number of significantly (>20%) increased metabolites that overlap between different conditions.

1. PCA loading plot


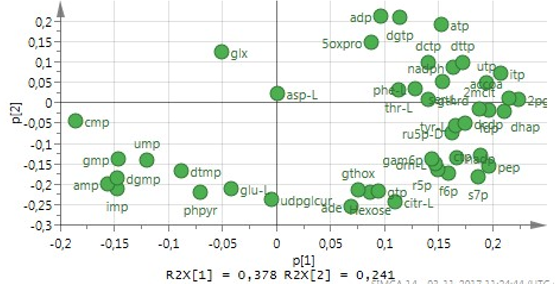


Additional file 1: Figure S5. Score plots of principal component analysis (PCA) of *P. taiwanensis* VLB120 metabolites under various inhibitory test.

1. **Heat map based on LC-MS data**


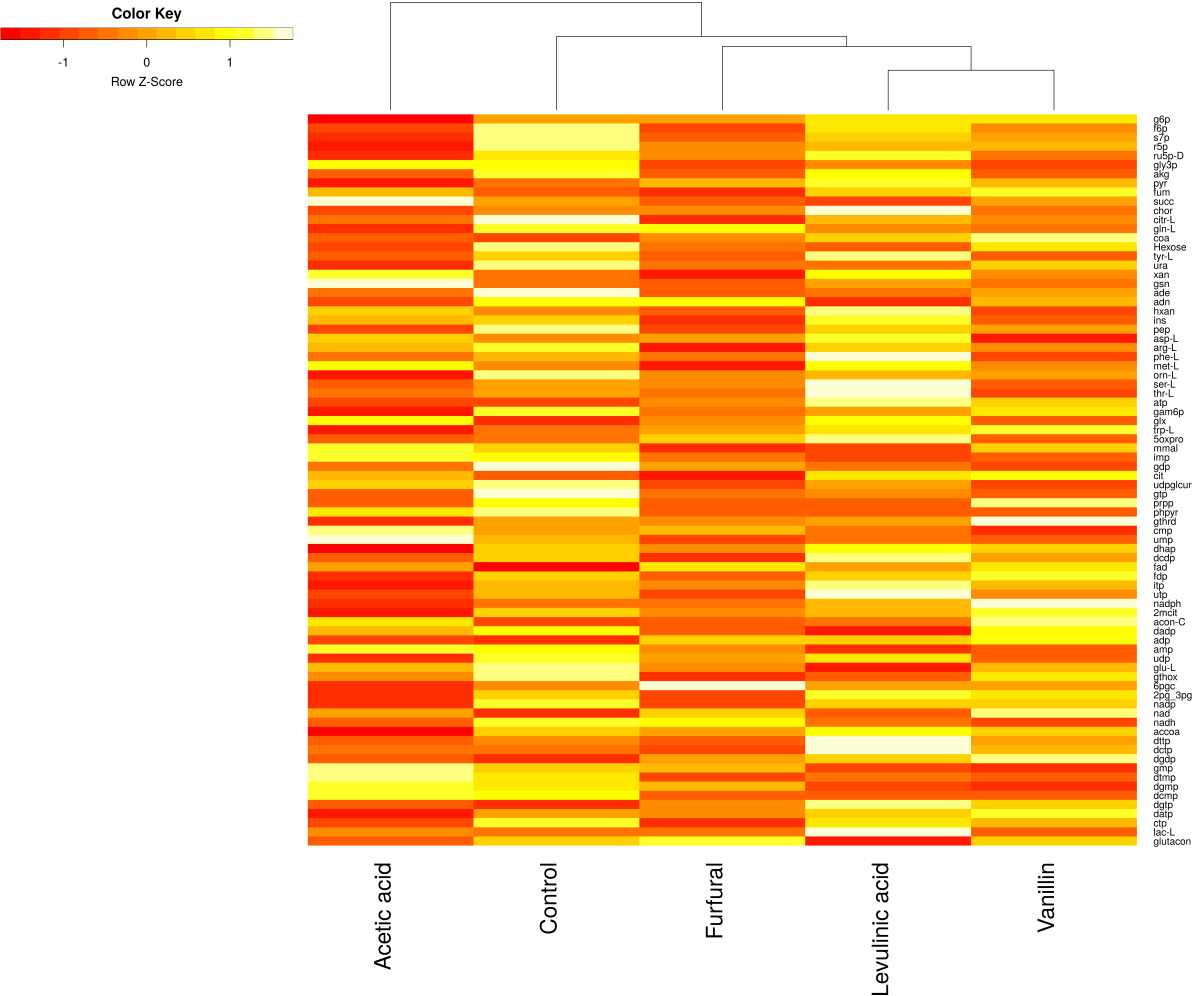


Additional file 1: Figure S6. Heatmap of metabolites extracted from *P. taiwanensis* VLB120 under different stress conditions. Metabolites abundance differences were clustered according to trends measured across all biological replicates (n = 3).
